# 

**Published:** 1883-05

**Authors:** 


CONTENTS:

| | PAGE. |
|---|---|
| ORIGINAL COMMUNICATIONS: | |
| Gangrene of the Leg, The Result of Injury—Fractures of the Femur—Hemorrhoids, Treatment by Ligature. By John Ashhurst, Jr., M. D., | 433 |
| An Experimental Study on the Action of Pilocarpin. By Dr. Julius Pohlman, | 441 |
| Tonsillotomy. By Charles C. F. Gay, M. D. | 446 |
| CLINICAL REPORTS: | |
| A Case of Diffuse Traumatic Cellulitis. By B. H. Daggett, M. D. | 450 |
| FOREIGN CORRESPONDENCE, | 453 |
| SELECTIONS: | |
| Radical Cure of Hernia, | 462 |
| Medical Corps U. S. Army, | 463 |
| Nitrous Oxide and Chloroform, | 464 |
| Glycerine, | 464 |
| Cæsarean Section, | 465 |
| SOCIETY REPORTS: | |
| Buffalo Medical and Surgical Association, | 465 |
| EDITORIAL CORRESPONDENCE: | |
| The Proposed State Board of Medical Examiners, | 470 |
| EDITORIAL: | |
| The Annual Report of the Buffalo State Asylum for the Insane, | 472 |
| The Profession or the Public, | 475 |
| Editorial Notes, | 477 |
| REVIEWS: | |
| The Tenth Annual Report of the Secretary of the State Board of Health of the State of Michigan, | 479 |
| A Manual of Auscultation and Percussion, Embracing the Physical Diagnosis of Diseases of the Lungs and Heart, and of Thoracic Aneurism, | 480 |
| Student's Guide to Diseases of the Eye, | 480 |

